# Activation of estrogen receptor β-dependent nitric oxide signaling mediates the hypotensive effects of estrogen in the rostral ventrolateral medulla of anesthetized rats

**DOI:** 10.1186/1423-0127-16-60

**Published:** 2009-07-07

**Authors:** Cheng-Dean Shih

**Affiliations:** 1 Department of Pharmacy & Graduate Institute of Pharmaceutical Technology, Tajen University, Pingtung 90741, Taiwan, Republic of China

## Abstract

**Background:**

Apart from their well-known peripheral cardiovascular effects, emerging evidence indicates that estrogen acts as a modulator in the brain to regulate cardiovascular functions. The underlying mechanisms of estrogen in central cardiovascular regulation, however, are poorly understood. The present study investigated the cardiovascular effects of 17β-estradiol (E2β) in the rostral ventrolateral medulla (RVLM), where sympathetic premotor neurons are located, and delineated the engagement of nitric oxide (NO) in E2β-induced cardiovascular responses.

**Methods:**

In male Sprague-Dawley rats maintained under propofol anesthesia, the changes of blood pressure, heart rate and sympathetic vasomotor tone after microinjection bilaterally into the RVLM of a synthetic estrogen, E2β were examined for at least 120 min. The involvement of ERα and/or ERβ subtypes was determined by microinjection of selective ERα or ERβ agonist into bilateral RVLM. Different NO synthase (NOS) inhibitors were used to evaluate the involvement of differential of NOS isoforms in the cardiovascular effects of E2β.

**Results:**

Bilateral microinjection of E2β (0.5, 1, or 5 pmol) into the RVLM dose-dependently decreased systemic arterial pressure (SAP) and the power density of the vasomotor components of SAP signals, our experimental index for sympathetic neurogenic vasomotor tone. These cardiovascular depressive effects of E2β (1 pmol) were abolished by co-injection of ER antagonist ICI 182780 (0.25 or 0.5 pmol), but not a transcription inhibitor actinomycin D (10 nmol). Like E2β, microinjection bilaterally into the RVLM of a selective ERβ agonist 2,3-*bis*(4-hydroxyphenyl) propionitrile (DPN, 1, 2, or 5 pmol) induced significant decreases in these hemodynamic parameters in a dose-dependent manner. In contrast, the selective ERα agonist 1,3,5-*tris*(4-hydroxyphenyl)-4-propyl-1H-pyrazole (5 pmol) did not influence the same cardiovascular parameters. Co-administration bilaterally into the RVLM of NOS inhibitor *N*^G^-nitro-L-arginine methyl ester (5 nmol) or selective inducible NOS (iNOS) inhibitor S-methylisothiourea (25 pmol), but not selective neuronal NOS inhibitor 7-nitroindazole (0.5 pmol) or endothelial NOS inhibitor N5-(1-Iminoethyl)-L-ornithine (2.5 pmol), significantly attenuated the cardiovascular depressive effects elicited by DPN (2 pmol).

**Conclusion:**

Our results indicate that E2β in the RVLM elicited short-term cardiovascular depressive effects via an ERβ-dependent nontranscriptional mechanism. These vasodepressor effects of E2β are likely to be mediated by the iNOS-derived NO in the RVLM.

## Background

Most known biological effects of estrogen, a major female gonadal steroid, are mediated by binding of the hormone to estrogen receptor (ER) subtypes, ERα and ERβ [1]. The ERs belong to a member of the nuclear hormone family of intracellular receptors that function as ligand-dependent transcriptional coactivators [1]. Upon binding to nuclear ERs, estrogen and ER forms a complex to bind at specific response elements in the promoters of target genes where it regulates gene transcription through direct interactions with DNA or other transcriptional machinery proteins [1,2]. This transcriptional mode of action is responsible for the well defined, long-lasting cellular responses of estrogen.

Recent studies have demonstrated that activated ER may promote physiological functions via nontranscriptional mechanisms [1,3,4]. In the heart and vasculature, the nongenomic mechanisms underlie estrogen-induced short-term arterial vasodilation [4,5], inhibition of atherosclerotic lesions [4,6] and amelioration of ischemia/reperfusion-induced cardiac injury [7]. These estrogen-triggered rapid cardiovascular responses are thought to be mediated via direct activation by the hormone of the cellular membrane rather than intracellular receptors [3,4,8].

Apart from their well-known peripheral cardiovascular effects, emerging evidence indicates that estrogen acts as a modulator in the brain to regulate cardiovascular functions [9-14]. Intracerebroventricular injection of estrogen increases systemic arterial pressure (SAP) and sympathetic nerve activity in female rats [9]. In estrogen-replaced ovariectomized female rats [12], but not in male rats [11], peripheral injection of estrogen decreases baseline sympathetic tone and SAP, which are antagonized by central injection of the selective ER antagonist [11,12]. Within the brain stem, both ER mRNA [15] and protein [16,17] are distributed in neurons of the rostral ventrolateral medulla (RVLM), where sympathetic premotor neurons for the maintenance of basal vasomotor tone are located [18], making this nucleus a candidate substrate to subserve the central cardiovascular regulatory actions of estrogen. Only a few studies [13,14] reported the cardiovascular regulatory actions of estrogen in the RVLM. Moreover, the underlying mechanisms mediating central cardiovascular effects of estrogen are not fully understood. The present study was therefore undertaken to evaluate the hypothesis that estrogen in the RVLM participates in neural regulation of cardiovascular functions via ERβ-dependent mechanisms that entail activation of nitric oxide (NO) signaling.

## Methods

### Animals

Experiments were carried out in adult male Sprague-Dawley rats (250–300 g, n = 126) purchased from the Experimental Animal Center of the National Science Council (Taipei, Taiwan). The male rats were chosen in this study to avoid possible confounding influence from differing circulating estrogen levels in cycling female rats. All rats were kept under conditions of constant temperature (23 ± 0.5°C) with a standard 12 h light-dark cycle (08:00–20:00) and free access to standard laboratory rat chow (Purina) and tap water. They were allowed to acclimatize for at least 7 days before experimental manipulations. All experiment procedures were conducted in compliance with the guidelines of our institutional animal care committee. All efforts were made to reduce the numbers of animals used and to minimize animal suffering during the experiment.

### General animal preparation

The preparatory surgery including intubation of the trachea and cannulation of the femoral artery and both femoral veins was performed under an induction dose of pentobarbital sodium (50 mg/kg, i.p.) [19,20]. During recording session, the anesthetic maintenance of the animals was achieved by an intravenous infusion of propofol (Zeneca Pharmaceuticals, Macclesfield, UK) at 20–30 mg/kg/h. This management scheme [21] provides satisfactory anesthetic maintenance while preserving the capacity of neural control of cardiovascular functions. The same management scheme also has no significant effect in increasing respiratory resistance [22] or airway mucus secretion [23]. Animals also received neuromuscular blockade with intravenous infusion of pancuronium (2 mg/kg/h) via the femoral vein. Pulsatile and mean SAP (MSAP), as well as heart rate (HR), was recorded on a polygraph (Gould RS3400, Valley View, OH, USA). Animals were ventilated mechanically by the use of a small rodent ventilator (Harvard 683, South Natik, MA, USA) to maintain an end-tidal CO_2 _within 4.0–4.5%, as monitored by a capnograph (Datex Normocap, Helsinki, Finland). This procedure was conducted to minimize possible confounding cardiovascular changes secondary to respiratory perturbation. The head of the animal was thereafter fixed to a stereotaxic headholder (Kopf 1430, Tujunga, CA, USA), and the rest of the body was placed on a thermostatically controlled pad to maintain rectal temperature of 37 ± 0.5°C. All data were collected from animals with a steady baseline MSAP above 90 mmHg throughout the recording period.

### Recording and power spectral analysis of SAP signals

The SAP signals recorded from the femoral artery were simultaneously subject to on-line power spectral analysis as detailed previously [21,24]. We were particularly interested in the very low-frequency (0–0.25 Hz) and low-frequency (0.25–0.8 Hz) components of the SAP spectrum. These spectral components of SAP signals were reported to take origin from the RVLM [25] and their power density reflect the prevailing sympathetic neurogenic vasomotor tone [19-21,26]. The power densities of these two spectral components were displayed during the experiment, alongside SAP, MSAP and HR, in an online and real-time manner.

### Microinjection of test agents into the RVLM

Microinjection bilaterally of test agents into the functionally identified RVLM sites were performed stereotaxically and sequentially with a glass micropipette (external tip diameter: 50–80 μm) connected to a 0.5-μl Hamilton microsyringe (Reno, NV, USA). A total volume of 50 nl was delivered to each side over 1–2 min to allow for complete diffusion of the injected solution. The time between injections from one side of the RVLM to the other was 5–6 min. The stereotaxic coordinates for the RVLM were 4.5 to 5.0 mm posterior to lambda, 1.8 to 2.1 mm lateral to midline, and 8.0 to 8.5 mm below the dorsal surface of the cerebellum. These coordinates were selected to cover the ventrolateral medulla in which both ER mRNA [15] and protein [16,17] are distributed, and where functionally identified sympathetic premotor neurons are located [18].

At the beginning of each experiment, the functional location of RVLM neurons on either side was established by monitoring a transient pressor response (15–25 mmHg) after microinjection of L-glutamate (1 nmol, Sigma Chemical). Subsequent microinjections of test agents were delivered to the identified pressor loci 20 min after the completion of glutamate application. This time lag was introduced to ensure complete recovery from the glutamate-induced pressor response before microinjection bilaterally into the RVLM of test agents or vehicle.

All microinjection solutions contained 1% Evans blue to aid in subsequent histological verification of the injection site. Possible volume effect of microinjection was controlled by injecting the same amount of artificial cerebrospinal fluid (aCSF, pH 7.4) of the following composition (in mM): 126 NaCl, 2.5 KCl, 2 CaCl_2_, 1–2 MgCl_2_, 1.25 NaH_2_PO_4_, 26 NaHCO_3_, and 10 D-glucose. To avoid confounding effects of drug interactions, each animal received only one treatment of synthetic estrogen, selective ERα, ERβ agonist or vehicle, given alone or in combination with one test agent.

### Preparation of test agents

The test agents were used in this study included 17β-estradiol-3-sulphate sodium (E2β; Sigma-Aldrich, St. Louis, MO, USA); a selective ERα agonist, 1,3,5-*tris*(4-hydroxyphenyl)-4-propyl-1H-pyrazole (PPT; Tocris Cookson Inc., Bristol, UK); a selective ERβ agonist, 2,3-*bis*(4-hydroxyphenyl) propionitrile (DPN; Tocris Cookson); 17α-estradiol (E2α; Sigma-Aldrich); a nonspecific ER antagonist, ICI 182780 (Tocris Cookson); a selective ERα antagonist, methyl-piperidino-pyrazole (MPP; Tocris Cookson); a selective ERβ antagonist, R,R-tetrahydrochrysene (R,R-THC; Tocris Cookson); a nonselective NO synthase (NOS) inhibitor, *N*^G^-nitro-L-arginine methyl ester (L-NAME; Sigma-Aldrich); a selective inducible NOS (iNOS) inhibitor, S-methylisothiourea (SMT; Tocris Cookson); a selective neuronal NOS (nNOS) inhibitor, 7-nitroindazole (7-NI; Tocris Cookson); a selective endothelial NOS (eNOS) inhibitor, N5-(1-Iminoethyl)-L-ornithine (L-NIO; Tocris Cookson); or a transcription inhibitor, actinomycin D (AMD; Tocris Cookson). The dose and treatment scheme were adopted from our preliminary experiments and previous studies [20,26], which used the same test agents for the same purpose as in this study. Moreover, to avoid confounding cardiovascular effects evoked by individual test agent, we purposely selected dose that did not alter the baseline circulatory parameters when microinjected alone into the bilateral RVLM. The dose of each antagonist or inhibitor used in this study, nonetheless, has been shown in our pilot studies to significantly inhibit cardiovascular responses induced by its specific ligand. All test agents were dissolved in aCSF at pH 7.4, with the exception of ICI 182780 and 7-NI, which used, respectively, 5% dimethyl sulfoxide (DMSO) or 3% methanol as the solvent. Control experiments showed that these vehicles had no significant effect on baseline MSAP or HR during the 120 min observation period.

### Brain histology

At the conclusion of each experiment, the animal was killed by an overdose of pentobarbital sodium, and the brain stem was removed from animals and fixed in 30% sucrose in 10% formaldehyde-saline solution for at least 72 h. Histological verification of the location of microinjection sites was carried out on frozen 25-μm sections of the medulla oblongata stained with 1% Neutral red.

### Statistical analysis

All values are expressed as mean ± SEM. The two-way analysis of variance (ANOVA) with repeated measures was used to assess group difference in the effect of various treatments on time course of changes in MSAP, HR or power density of vasomotor components of SAP spectrum. This was followed by the Scheffé multiple-range test for *post hoc *assessment of individual means. The maximal changes in the hemodynamic parameters were evaluated with paired *t*-test. *P *< 0.05 was considered statistically significant.

## Results

### Cardiovascular effects of microinjection bilaterally into the RVLM of E2β

**Figure 1:**
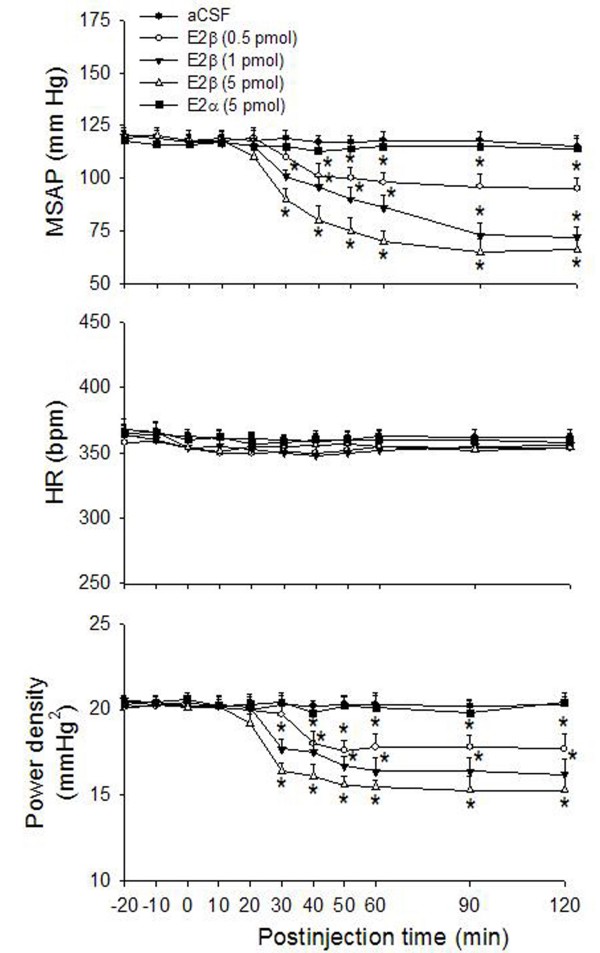
**Cardiovascular effects after microinjection bilaterally into the RVLM of E2β**. Time-course of the changes in mean systemic arterial pressure (MSAP), heart rate (HR) and total power density of vasomotor components (0–0.8 Hz) of systemic arterial pressure (SAP) spectrum in anaesthetized rats that received microinjection bilaterally into the rostral ventrolateral medulla (RVLM, at time 0) of artificial cerebrospinal fluid (aCSF), 17β-estradiol (E2β, 0.5, 1 or 5 pmol), or 17α-estradiol (E2α, 5 pmol). Values are presented as mean ± S.E.M., n = 6–8 animals per experimental group. *P < 0.05 versus corresponding aCSF group in the Scheffé multiple range test.

Compared to aCSF treatment, microinjection bilaterally into the functionally identified pressor region of RVLM of E2β (0.5, 1, or 5 pmol) resulted in significant and dose-dependent decreases in MSAP and power density of vasomotor components of SAP spectrum, our experimental index for sympathetic neurogenic vasomotor outflow [19-21,24], without apparent effect on HR (Fig. 1). The cardiovascular depressive responses of E2β commenced approximately 30 min, and lasted for at least 120 min posttreatment. Duration of cardiovascular depressive responses of E2β (0.5, 1, or 5 pmol) was dose dependent. At a lower dose (0.5 pmol), E2β promoted vasodepressor responses that lasted for approximately 150 min postinjection, whereas at higher doses (1 or 5 pmol) E2β produced cardiovascular depressive responses that sustained more than 3–4 hrs postinjection. Microinjection bilaterally into the RVLM of the inactive isomer of estrogen, E2α (5 pmol), on the other hand, did not affect basal hemodynamic parameters (Fig. 1). Microinjection of E2β (5 pmol) into the ventrolateral medullary areas adjacent to, but outside the confine of RVLM, e.g., spinal trigeminal nucleus or lateral paragigantocellular nucleus, also elicited minimal effects on those cardiovascular parameters (data not shown). In addition, intravenous injection of a high dose (5 pmol) E2β did not affect baseline MSAP, HR or power density of vasomotor components of SAP spectrum (MSAP: -3.2 ± 1.6 mmHg; HR: +4.3 ± 0.8 bpm; SAP spectrum: -0.6 ± 0.5 mmHg^2^, n = 3).

### Effects of ER antagonist on the E2β-induced cardiovascular depressive responses

**Figure 2:**
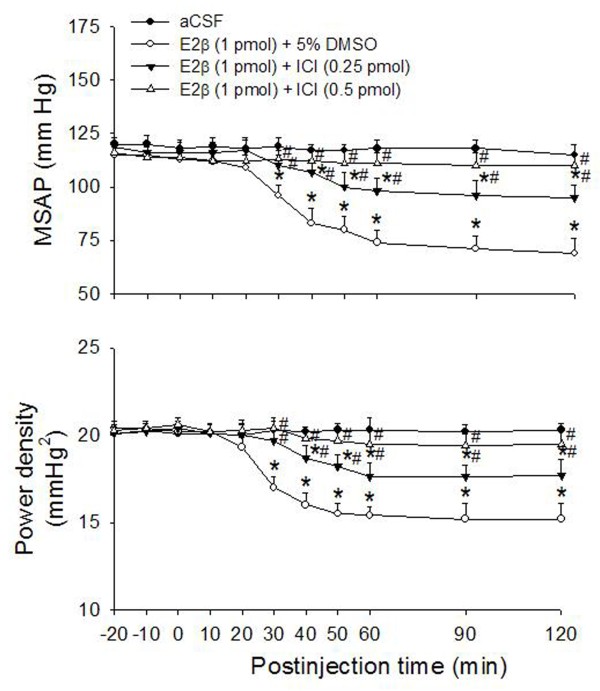
**Effects of ER antagonist on the E2β-induced cardiovascular depressive responses**. Time-course of the changes in MSAP and total power density of vasomotor components (0–0.8 Hz) of SAP spectrum in anaesthetized rats that received microinjection bilaterally into the RVLM (at time 0) of aCSF, or E2β (1 pmol) given together with ICI 182780 (ICI, 0.25 or 0.5 pmol) or 5% DMSO. Values are presented as mean ± S.E.M., n = 6–8 animals per experimental group. *P < 0.05 versus corresponding aCSF group, and ^#^P < 0.05 versus corresponding E2β+DMSO group in the Scheffé multiple range test.

Co-administration bilaterally into the RVLM of a nonspecific ER antagonist, ICI 182780 (0.25 or 0.5 pmol) attenuated the cardiovascular depressive responses of E2β (1 pmol) (Fig. 2) in a dose-related manner. At high dose (0.5 pmol), ICI 182780 almost completely reversed the E2β-induced hypotension and the decrease in sympathetic vasomotor tone. Comparable results were obtained in animals that received ICI 182780 delivered at 20 min before E2β microinjection (data not shown). In addition, co-administration bilaterally into the RVLM of a specific ERβ antagonist R,R-THC (50 pmol), but not a specific ERα antagonist MPP (1 nmol), attenuated the vasodepressor responses induced by E2β (1 pmol) (maximal decrease in MSAP: E2β alone: -48.6 ± 6.3 mmHg vs. E2β+R,R-THC: -18 ± 6.1 mmHg, n = 4, *P *< 0.05; maximal decrease in MSAP: E2β alone: -48.6 ± 6.3 mmHg vs. E2β+MPP: -50.3 ± 5.4 mmHg, n = 4, *P *> 0.05). Microinjection bilaterally into the RVLM of ICI 182780 (0.25 or 0.5 pmol), R,R-THC (50 pmol) or MPP (1 nmol) alone, on the other hand, had no discernible effect on baseline MSAP, HR or power density of vasomotor components of SAP spectrum (Table 1). In contrast, co-administration bilaterally into the RVLM of a transcription inhibitor AMD (10 nmol), did not affect the cardiovascular depressive response of E2β (1 pmol) (maximal decrease in MSAP: E2β alone: -46.8 ± 5.3 mmHg vs. E2β+AMD: -52 ± 6.5 mmHg, n = 6, *P *> 0.05).

**Table 1:** Effects of test agents on baseline MSAP, HR and power density of vasomotor components of SAP spectrum

*Treatment*	*Maximal changes in*		
	
	*MSAP (mmHg)*	*HR (bpm)*	*Power Density (mmHg*^2^)
aCSF	+3.3 ± 0.4	+4.9 ± 0.6	+0.7 ± 0.5
ICI 182780 (0.25 pmol)	+3.3 ± 0.6	+5.5 ± 0.8	+0.7 ± 0.5
ICI 182780 (0.5 pmol)	+2.5 ± 0.5	+3.5 ± 0.8	+0.6 ± 0.6
R,R-THC (50 pmol)	+6.4 ± 0.6	+6.5 ± 1.0	+0.8 ± 0.4
MPP (1 nmol)	+6.1 ± 0.8	+6.6 ± 0.8	+0.9 ± 0.7
L-NAME (5 nmol)	+3.8 ± 0.6	+5.6 ± 0.6	+0.9 ± 0.6
SMT (25 pmol)	+3.7 ± 0.8	+5.3 ± 0.8	+0.7 ± 0.5
7-NI (0.5 pmol)	-2.7 ± 0.8	-3.3 ± 0.5	-0.6 ± 0.8
L-NIO (2.5 pmol)	-1.9 ± 0.8	-3.6 ± 0.7	-1.0 ± 0.6

Values are mean ± S.E.M., n = 6–7 animals per experimental group. No significant difference was detected among groups in the paired *t*-test.

### Cardiovascular effects of microinjection bilaterally into the RVLM of ERα or ERβ agonist

**Figure 3:**
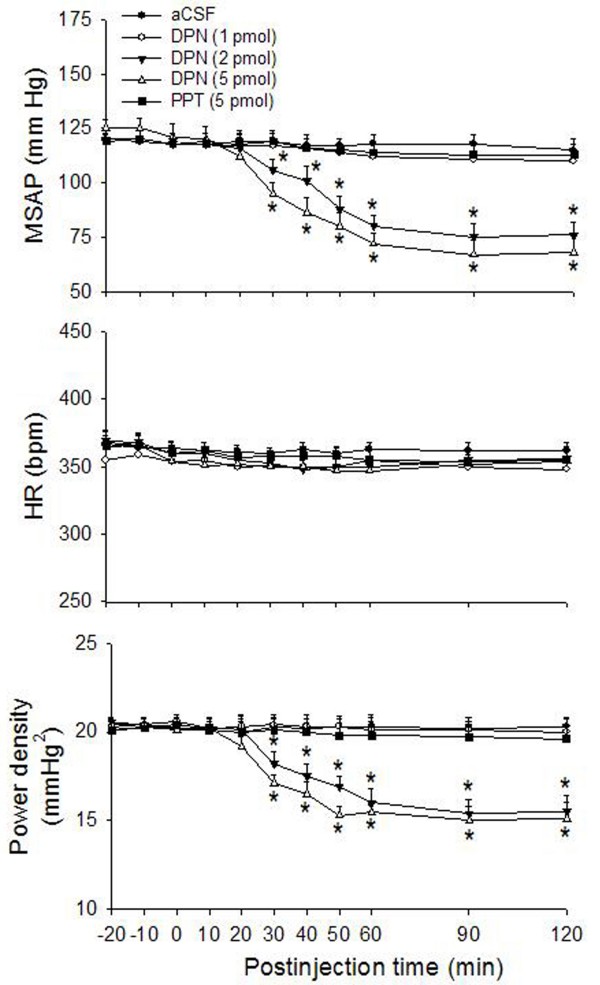
**Cardiovascular effects after microinjection bilaterally into the RVLM of ERα or ERβ agonist**. Time-course of the changes in MSAP, HR and total power density of vasomotor components (0–0.8 Hz) of SAP spectrum in anaesthetized rats that received microinjection bilaterally into the RVLM (at time 0) of aCSF, DPN (1, 2 or 5 pmol), or PPT (5 pmol). Values are presented as mean ± S.E.M., n = 6–8 animals per experimental group. *P < 0.05 versus corresponding aCSF group in the Scheffé multiple range test.

To decipher the role of ERα and/or ERβ subtypes in E2β-induced cardiovascular depressive responses, we evaluated the cardiovascular effects of the selective ERα or ERβ agonist in the RVLM. Similar to effects induced by E2β, microinjection bilaterally into the RVLM of ERβ agonist DPN (1, 2 or 5 pmol) promoted hypotension and reduction in sympathetic vasomotor tone in a dose-related manner (Fig. 3). In contrast, the same doses of DPN (2 or 5 pmol) microinjected outside the confine of RVLM (e.g., spinal trigeminal nucleus or lateral paragigantocellular nucleus) caused a minimal alteration in the same cardiovascular parameters (data not shown). In a separate series of experiments, microinjection bilaterally into the RVLM of ERα agonist PPT (5 pmol) did not affect basal hemodynamic parameters (Fig. 3). Similarly, intravenous injection of DPN (5 pmol) did not affect the baseline MSAP, HR or power density of vasomotor components of SAP spectrum (MSAP: -4.1 ± 1.2 mmHg; HR: +4.4 ± 1.1 bpm; SAP spectrum: -0.7 ± 0.5 mmHg^2^, n = 3).

### Effects of NOS inhibitor on the ERβ agonist-induced cardiovascular depressive effects

**Figure 4:**
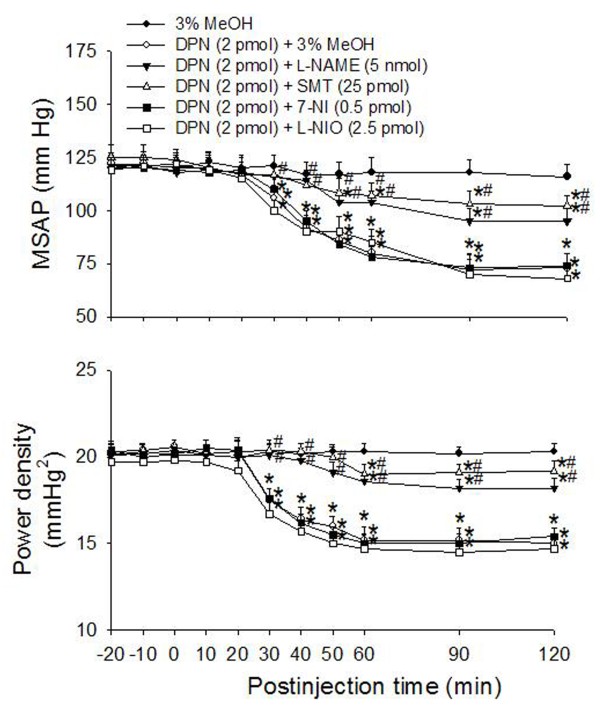
**Effects of NOS inhibitor on the ERβ agonist-induced cardiovascular depressive effects**. Time-course of the changes in MSAP and total power density of vasomotor components (0–0.8 Hz) of SAP spectrum in anaesthetized rats that received microinjection bilaterally into the RVLM (at time 0) of aCSF, 3% methanol (MeOH), or DPN (2 pmol) given together with L-NAME (5 nmol), SMT (25 pmol), 7-NI (0.5 pmol), L-NIO (2.5 pmol) or 3% MeOH. Values are mean ± S.E.M., n = 6–7 animals per experimental group. *P < 0.05 versus corresponding MeOH group, and ^#^*P *< 0.05 versus corresponding DPN+MeOH group in the Scheffé multiple range test. Data on aCSF are not shown because they in essence duplicated those by 3% MeOH.

Compared with the aCSF or 3% MeOH controls, co-administration of the nonselective NOS inhibitor, L-NAME (5 nmol) significantly attenuated hypotension and reduction in power density of vasomotor components of SAP spectrum promoted by microinjection bilaterally into the RVLM of DPN (2 pmol) (Fig. 4). Of the three isoforms of NOS, we found that only the iNOS inhibitor SMT (25 pmol), but not the nNOS inhibitor 7-NI (0.5 pmol) or the eNOS inhibitor L-NIO (2.5 pmol), appreciably attenuated the DPN-induced cardiovascular depressive responses (Fig. 4). Microinjection into bilateral RVLM of L-NAME (5 nmol), SMT (25 pmol), 7-NI (0.5 pmol), or L-NIO (2.5 pmol) alone had no significant effect on baseline MSAP, HR or power density of vasomotor components of SAP spectrum (Table 1).

### Microinjection sites

**Figure 5:**
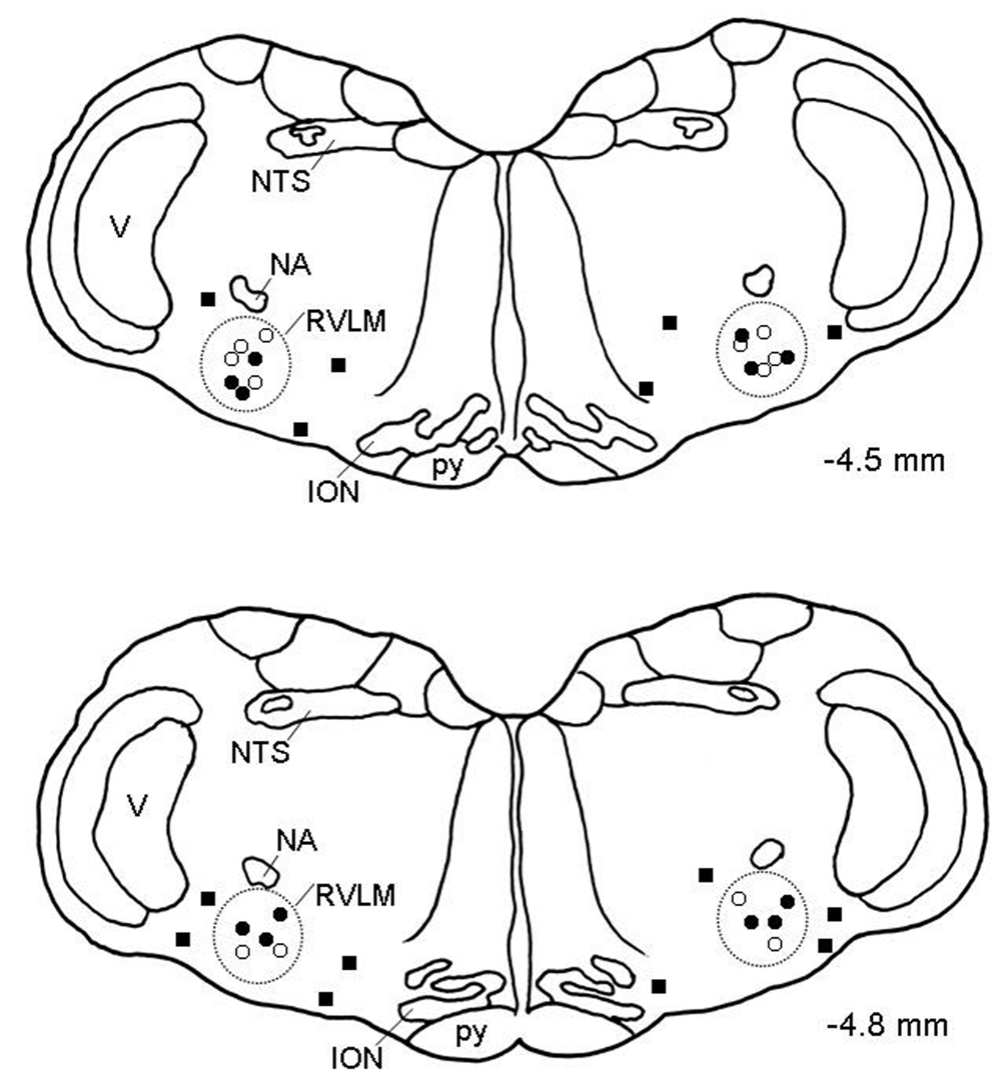
**Microinjection sites of E2β and DPN in the RVLM**. Diagrammatic representations of two rostral-caudal levels of the RVLM (dotted line areas) with reference to the lambda illustrating the location of sites where bilateral microinjection of E2β (black circle, 5 pmol) and DPN (◦,5 pmol) elicited significant inhibitory or minimal effects (black square, non-RVLM microinjection sites) on the MSAP and power density of vasomotor components of SAP spectrum. Numbers on right side indicate distance from the lambda. For the purpose of clarity, approximately 20% of the total microinjection sites are included and are presented on both side of the diagram. ION, inferior olivary nucleus; NA, nucleus ambiguous; NTS, nucleus tractus solitarii; RVLM, rostral ventrolateral medulla; V, nucleus of the spinal trigeminal nerve; py, pyramidal tract.

Histological verification of locations of micropipette tips in the ventrolateral medulla confirmed that all observations were made from animals that received local administration of the test agents within the anatomic confines of the RVLM (Fig. 5). For the purpose of clarity, Fig. 5 only summarizes the location of sites where microinjection of E2β (5 pmol) and DPN (5 pmol) elicited significant (*P *< 0.05) inhibitory effects on the MSAP and power density of vasomotor components of SAP spectrum.

## Discussion

To the best of our knowledge, the present study is the first demonstration of an active role of ERβ at the RVLM in central cardiovascular regulation. We found that E2β and ERβ agonist dose-dependently decreased MSAP and power density of vasomotor components of SAP spectrum, whereas ERα agonist did not change these hemodynamic parameters. We further demonstrated that the iNOS-derived NO in the RVLM is involved in the ERβ-dependent cardiovascular depressive effects.

Accumulative evidence indicates estrogen as an active hormone in central cardiovascular regulation [9-14] via activation of the ERα and ERβ [1]. Within the brain stem, ERα and ERβ mRNA [15] as well as protein [16,17]are expressed in the RVLM. Function of these RVLM neurons expressing ERs, however, is not fully understood. One major finding of the present study is the identification of a short-term vasodepressor effect of estrogen in the RVLM. A lower dose (0.5 pmol) of E2β promoted acute vasodepressor responses that lasted for 150 min postinjection, whereas at higher doses (1 or 5 pmol) E2β produced similar effects that sustained more than 3–4 hrs postinjection. It has been reported that direct microinjection of the same steroid hormone (0.5 μM) into the RVLM produces a short-term (<60 min) hypotension [13]. These results suggest that the duration of short-term vasodepressor effects of E2β in the RVLM is manifested in a dose-dependent manner. Long-term (>24 hrs) cardiovascular effect of E2β in the RVLM and the involvement of nongenomic and/or genomic signaling mechanisms, however, await further investigation. In addition, we demonstrated that these cardiovascular regulatory effects of the female steroids are nucleus specific, since application of E2β or its agonist, DPN, to areas outside the confines of the RVLM did not influence the baseline hemodynamics. More importantly, we found that high dose E2β or DPN promoted cardiovascular depression only when they were microinjected into the bilateral RVLM but not intravenously. These findings confirmed that the E2β- and DPN-induced cardiovascular depressive responses are not caused by leakage of these test agents from the injection site in the brain to the peripheral circulation.

There are only a limited data from in vivo studies to demonstrate the role of estrogen in central cardiovascular regulation. Consistent with our observation, a previous study [13] reported that direct microinjection of E2β into the RVLM elicits significant decreases in SAP and sympathetic nerve activity in male rats, suggesting that estrogen participate in central cardiovascular regulation by acting directly on the RVLM. At receptor level, we found that the cardiovascular depressive effects of E2β are mediated via activation of the ERβ in the RVLM. The nonselective ER antagonist ICI 182780, at the dose that did not by itself alter the baseline circulatory parameters implying that endogenous estrogen may not exerts a tonic effect on the cardiovascular responses at the level of the RVLM, whereas this antagonist almost completely reversed the cardiovascular depressive effects by E2β. Furthermore, we also demonstrate that the cardiovascular depressive effects induced by selective ERβ agonist DPN, but not the selective ERα agonist PPT, are similar to that induced by E2β. We also employed specific ERα or ERβ antagonist to demonstrate engagement of ERβ but not ERα in the E2β-mediated vasodepressor effects in the RVLM. These results suggest that E2β in the RVLM induces vasodepressor effects mainly via the ERβ-mediated mechanisms. This suggestion is supported by the study that in the isolated RVLM neurons, E2β possesses rapid inhibitory effect on the voltage-gated Ca^++ ^currents, which is mimicked by the ERβ-selective but not the ERα-selective agonist [17]. Additionally, ERβ knockout mice exhibit sustained hypertension and abnormal vascular function indicating an essential role for ERβ in the regulation of vascular function and blood pressure [27]. Anatomically, the distribution of ERβ-immunoreactivity in the RVLM neurons is prominent in the extra-nuclear sites than that of ERα, particularly on plasma membranes [17]. The observed short-term hypotension and reduction in sympathetic vasomotor tone, which occurred approximately 30 min after microinjection of E2β or ERβ agonist into the RVLM, may therefore mediated by a nongenomic signaling mechanism. In support of this suggestion, we found that transcription inhibitor, AMD, did not affect the E2β-induced short-term vasodepressor response. The rapid nongenomic actions of estrogen in both cardiac [7,28] and vascular [4,5] systems are mediated directly by ERs located in or close to the cellular membrane rather than the nuclear sites [3,4,8]. Cellular mechanism underling the ERβ-mediated short-term cardiovascular effect in the RVLM, however, awaits further investigation.

Another major contribution of this study is to demonstrate the involvement of NO in cardiovascular depression induced by activation of ERβ in the RVLM. NO is a well-established neuromodulator for central cardiovascular regulation in the RVLM [26,29-34]. Microinjection of the NO precursor or donor into the RVLM reportedly produces prominent depressor effects and reduced sympathetic nerve activity [26,29-31]. In the present study we found that the ERβ agonist-induced cardiovascular depressive responses were attenuated by co-administration of the nonselective NOS inhibitor, L-NAME. These results indicate that NO is engaged in cardiovascular depression induced by activation of ERβ and further imply that cardiovascular depressive responses of estrogen may be mediated via an interaction between estrogen and NO signals in the RVLM. This suggestion is supported by the study that estrogen alters the NOS expression via activation of ERβ in the hypothalamic paraventricular nucleus [35]. In addition, estrogen replacement in ovariectomized rats reduces arterial pressure responses to psychological stress by increasing NO production in brain stem and hypothalamus [36]. Intracarotid injection of estrogen inhibits spontaneous electrical activity of RVLM neurons via the activation of NO-dependent signaling pathway [37]. Of the three isoforms of NOS that have been identified in the RVLM, we found that NO derived from iNOS may play a major role in the ERβ-dependent cardiovascular depression. The iNOS-derived NO in the RVLM has been reported to elicit sympathoinhibition and vasodepressor effects [26,33,34]. A negative result of 7-NI or L-NIO, on the other hand, implies a minor role of nNOS and eNOS in the ERβ-dependent cardiovascular depression in the RVLM. At a lower dose than those used in the present study, 7-NI or L-NIO has been reported to selective inhibit the nNOS-derived NO in the hypothalamic paraventricular nucleus [38] or L-arginine-induced NO synthesis in the vascular endothelium [39]. We noted that L-NAME or iNOS inhibition did not completely attenuate the cardiovascular depressive responses induced by ERβ agonist. The involvement of NO-independent alternative pathways in the cardiovascular depressive effects of E2β in the RVLM, therefore, awaits further investigation.

To avoid confounding cardiovascular effects caused by differing levels of circulating estrogen through various stages in cycling female rats, we purposely selected male animals in the present study. In addition, a number of previous studies on the rapid cardiovascular effects of estrogen in the RVLM [13] or other central autonomic nuclei [13,40] were done in male rats. PPT and DPN are reported to be a useful experimental tool to study the differences in structure and biological functions of ERα and ERβ [17,41,42]. The selectivity of nonsteroidal estrogen, PPT and DPN as respective ERα and ERβ agonist has been documented [17,41,42]. PPT and DPN exhibits a relative high selectivity toward ERα and ERβ, respectively, when compared with some phytoestrogens such as genistein and coumestrol [43]. We also realize that this study was conducted under an anesthetic condition that may be a major confounding factor to the observed cardiovascular responses. This possibility, however, is deem unlikely since we demonstrated previously [19,20] that the anesthetic maintenance scheme (i.e., propofol at 20–30 mg/kg/h) used in this study has no discernible effect on the sympathetic vasomotor outflow from the RVLM, and hence baseline MSAP and HR [21].

In conclusion, our results demonstrate that the E2β in the RVLM elicited short-term non-genomic cardiovascular inhibitory effects via activation of ERβ. Furthermore, NO derived from iNOS may contribute to central vasodepressor effects after activation of ERβ in the RVLM.

## Competing interests

The author declares that they have no competing interests.

## Authors' contributions

CDS participated in experimental conception and design, performed animal and pharmacological experiments, acquisition of data, the statistical analysis and interpretation of data, and was also involved in drafting and revising the manuscript and have given final approval of the version to be published.

Author read and approved the final manuscript.
